# The functions and inhibitors of protein tyrosine phosphatase B from *Mycobacterium tuberculosis*

**DOI:** 10.3389/fimmu.2026.1833963

**Published:** 2026-06-04

**Authors:** Haonan Wang, Leiliang Zhang

**Affiliations:** 1 Department of Clinical Laboratory Medicine, The First Affiliated Hospital of Shandong First Medical University and Shandong Provincial Qianfoshan Hospital, Jinan, Shandong, China; 2 Department of Pathogen Biology, School of Clinical and Basic Medical Sciences, Shandong First Medical University and Shandong Academy of Medical Sciences, Jinan, Shandong, China

**Keywords:** *Mycobacterium tuberculosis*, PtpB, PI(4,5)P2, gasdermin D, JAK-STAT

## Abstract

*Mycobacterium tuberculosis* (Mtb) secretes the low molecular weight protein tyrosine phosphatase B (PtpB), which disrupts key host innate immune defenses. This review synthesizes current knowledge regarding PtpB, highlighting its unique structural features, including a ubiquitin-activated dynamic lid that regulates its catalytic activity toward diverse substrates. PtpB is known to suppress pro-inflammatory cytokine production by dephosphorylating essential components of the MAPK and JAK-STAT signaling pathways. Additionally, upon activation by host ubiquitin, PtpB directly dephosphorylates plasma membrane lipids such as phosphatidylinositol 4-phosphate (PI4P) and phosphatidylinositol 4,5-bisphosphate [PI (4,5)P2], thereby inhibiting the plasma membrane localization of gasdermin D and blocking pyroptosis. This review further evaluates the translational potential of targeting PtpB, emphasizing the discovery and structural characterization of potent, selective inhibitors. Ultimately, PtpB emerges as an attractive target for novel host-directed therapies against tuberculosis, especially in the face of rising drug resistance.

## 1 Introduction

Tuberculosis (TB), primarily caused by *Mycobacterium tuberculosis* (Mtb), remains one of the most significant global health challenges. According to the World Health Organization’s Global Tuberculosis Report 2024, although global TB incidence and mortality rates have shown a modest decline in recent years, indicating a recovery of essential health services following the COVID-19 pandemic, these gains are fragile and unevenly distributed (1). The path to ending TB is fraught with persistent challenges, including the rise of multidrug-resistant (MDR) and extensively drug-resistant (XDR) strains, significant funding gaps for prevention and research, and critical drivers such as undernutrition and comorbidities (2).

Upon infecting host macrophages, Mtb secretes a range of virulence factors that modulate host cell signaling to evade immune clearance (3). Among these effectors are protein tyrosine phosphatase A (PtpA), secreted acid phosphatase (SapM), and protein tyrosine phosphatase B (PtpB), which collectively disrupt key innate immune pathways. Genetic deletion of *PtpA*, *SapM*, or *PtpB* in Mtb results in reduced bacterial survival in macrophages and attenuated virulence in animal models, highlighting the important role these phosphatases play in intracellular persistence (3–7). Notably, although PtpB is consistently detected in the macrophage cytosol during Mtb infection, the exact molecular mechanism governing its secretion remains an open question in the field, hindering the development of targeted intervention strategies. Beresford et al. identified PtpB as a critical effector in mediating immune evasion (8). It is a low molecular weight protein tyrosine phosphatase with triple specificity, capable of dephosphorylating not only phosphotyrosine and phosphoserine/threonine residues on host proteins but also phosphoinositide lipids on the plasma membrane (8). Furthermore, studies by Chai et al. demonstrated that, upon activation by host ubiquitin, PtpB directly dephosphorylates phosphatidylinositol 4-phosphate (PI4P) and phosphatidylinositol 4,5-bisphosphate [PI (4,5)P2], effectively blocking gasdermin D (GSDMD)-mediated pyroptosis (9). Structural studies of these virulence factors have provided critical insights into their catalytic mechanisms and have opened new avenues for structure-based drug design.

The roles of mycobacterial secreted tyrosine phosphatases, particularly PtpA and PtpB, in undermining host immunity have been extensively reviewed, underscoring their evolution as key virulence factors and promising targets for host-directed therapy (10–15). Recent syntheses have consolidated structural and functional insights into these phosphatases, mapping the journey from mechanistic discovery to inhibitor development. Notably, the unique mechanism of PtpB in inhibiting pyroptosis through phosphoinositide dephosphorylation has been recognized as a significant advancement in understanding Mtb’s immune evasion strategies (16, 17). These foundational and contemporary reviews lay the groundwork for a focused examination of PtpB, its mechanisms, and the translational pursuit of its inhibitors.

Given its central role in immune evasion, as supported by genetic deletion studies in macrophages and animal models, along with the use of selective small-molecule inhibitors, PtpB has emerged as a promising preclinical target for novel anti-tuberculosis drugs (18–20). Intensive research has led to the discovery of potent and selective inhibitors, such as the natural product Kuwanol E and synthetic inhibitors (oxalylamino-methylene)-thiophene sulfonamide (OMTS), which effectively block PtpB’s phosphatase activity *in vitro* (19, 20). The crystal structures of PtpB, both in its auto-inhibited state and in complex with inhibitors, have unveiled unique structural features, such as a dynamic two-helix lid over the active site, that provide a blueprint for designing highly selective compounds (19, 21).

This review will synthesize current knowledge on PtpB, beginning with an analysis of its structure-function relationship, followed by a detailed discussion of its multifaceted roles in undermining host immunity. The review will conclude with an overview of the progress and challenges in developing PtpB inhibitors as next-generation therapeutics against TB.

## 2 The structural and biochemical basis of PtpB

**Figure 1:**
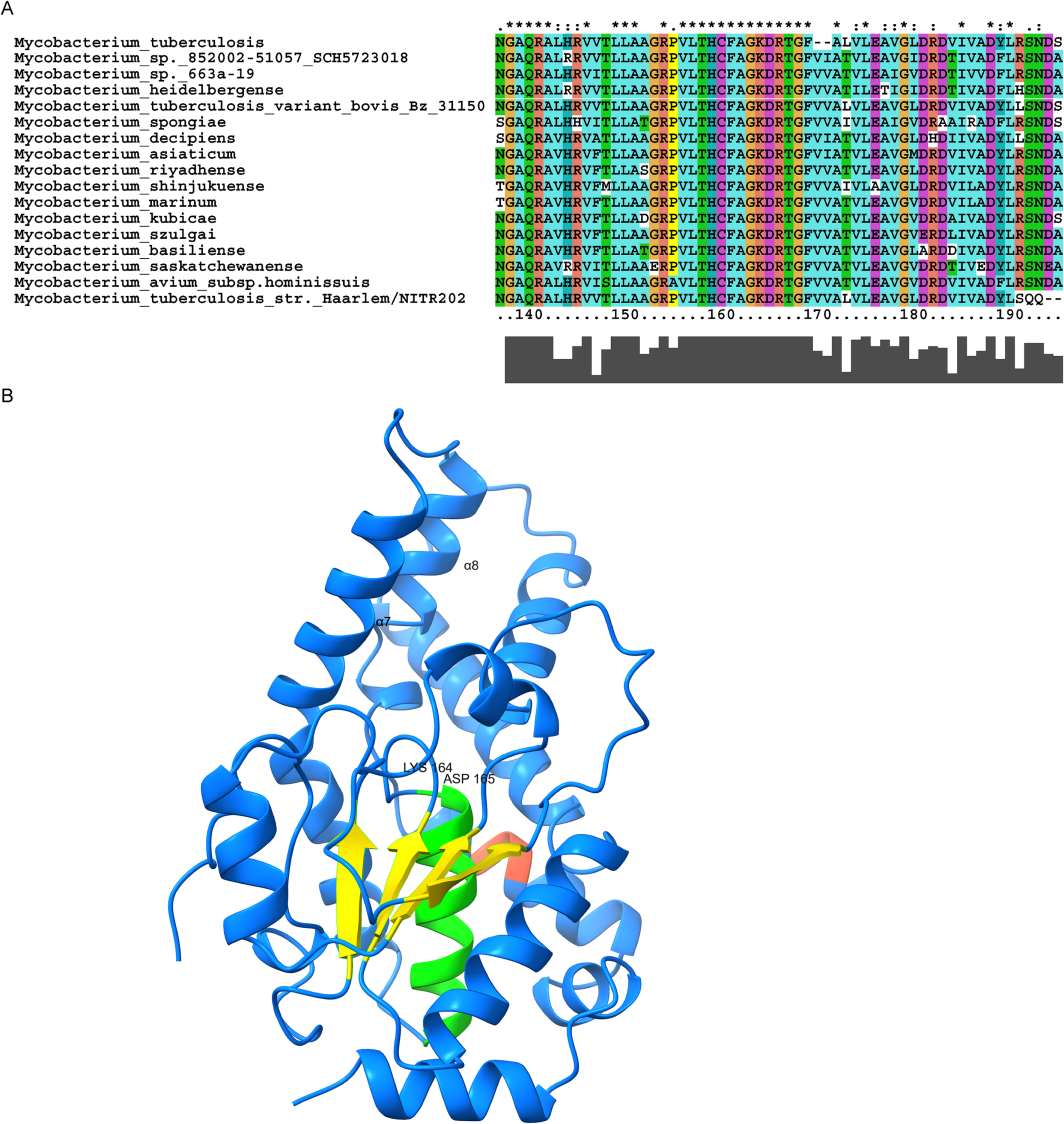
The structural and biochemical basis of PtpB. **(A)** Amino acid sequence alignment of PtpB orthologs from various Mycobacterium species was generated using ClustalX (version 2.1) and is adapted from the original analysis presented by Beresford et al. (8). *Mycobacterium_tuberculosis* (WP_057174265.1), *Mycobacterium_sp._852002-51057_SCH5723018* (WP_067114712.1), *Mycobacterium_sp._663a-19* (WP_324675357.1), *Mycobacterium_heidelbergense* (WP_083072897.1), *Mycobacterium_tuberculosis_variant_bovis_Bz_31150* (KAN85583.1), *Mycobacterium_spongiae* (WP_211697385.1), *Mycobacterium_decipiens* (WP_085324078.1), *Mycobacterium_asiaticum* (WP_065146116.1), *Mycobacterium_riyadhense* (WP_085252303.1), *Mycobacterium_shinjukuense* (WP_083046766.1), *Mycobacterium_marinum* (WP_261899836.1), *Mycobacterium_kubicae* (WP_186273621.1), *Mycobacterium_szulgai* (WP_085673027.1), *Mycobacterium_basiliense* (WP_158014975.1), *Mycobacterium_saskatchewanense* (WP_085254357.1), *Mycobacterium_avium_subsp.hominissuis* (GAB4942954.1), *Mycobacterium_tuberculosis_str._Haarlem/NITR202* (AGL21928.1). **(B)** Ribbon diagram of PtpB structure (PDB ID: 2OZ5) illustrating the central four-stranded β-sheet (orange), the buried active-site P-loop (green) and the UIM-like region (red).

The multifaceted role of PtpB in undermining host defense is fundamentally rooted in its unique molecular structure and precisely regulated enzymatic activity. As a secreted virulence factor of Mtb, PtpB exhibits triple-specificity phosphatase activity. It possesses a broad substrate profile that includes phosphotyrosine and phosphoserine/threonine residues on host signaling proteins, as well as phosphoinositide lipids on the plasma membrane. This functional versatility is attributed to a conserved catalytic core containing the P-loop motif CX5R, a hallmark of the protein tyrosine phosphatase (PTP) superfamily that is essential for coordinating and hydrolyzing phosphate groups from its diverse substrates (**Figure 1A**). As demonstrated by Bereford and colleagues, the active site features two key residues: Asp165, which is catalytically essential, and Lys164, which serves as a substrate-dependent specificity modulator (**Figure 1B**) (8). This capability to hydrolyze diverse substrates underpins PtpB’s role in Mtb virulence.

The three-dimensional structure of PtpB was first determined by Grundner and colleagues (21). PtpB is a single-domain, globular protein with a central, four-stranded parallel β sheet surrounded by α helices (**Figure 1B**). A defining structural characteristic of PtpB is a dynamic two-helix lid formed by α7 and α8 (residues 211-224) that covers the active site. In its closed state, the lid sterically blocks the catalytic pocket, maintaining PtpB in an auto-inhibited state. This is supported by its 1.7 Å crystal structure, which reveals that the lid seals the active site, rendering PtpB catalytically inactive. Furthermore, the lid contains Phe222, which, together with a bound phosphate mimic of a phosphotyrosine substrate, reinforces this inhibition. Activation of PtpB involves the rearrangement of this lid, a process triggered by specific interactions that open the active site for substrate access and catalysis. This gating mechanism ensures precise control of phosphatase activity while protecting the active site from oxidative inactivation (21).

Additionally, PtpB contains a ubiquitin-interacting motif (UIM)-like region, characterized by a hydrophobic surface involving residues Ala240-Ala242 on helix α9, which specifically engages the Ile44 residue of host-derived mono-ubiquitin (9). As demonstrated by Chai and colleagues (9), ubiquitin binding functions as an allosteric switch, releasing the lid and exposing the catalytic P-loop, thereby activating its phospholipid phosphatase function, which is critical for inhibiting pyroptosis as described in the subsequent section. The role of ubiquitin in regulating mycobacterial phosphatases appears to be complex. While the activation of PtpB by mono-ubiquitin binding to its UIM-like region is well-established in relation to its lipid phosphatase activity, reports on PtpA’s interaction with ubiquitin remain contradictory. Some studies suggest that PtpA is also activated by ubiquitin (22), while others present conflicting evidence (23). This highlights the need to clarify whether ubiquitin-mediated regulation is a general feature of mycobacterial phosphatases or specific to PtpB, which has significant implications for designing selective inhibitors that disrupt this host-pathogen interface.

This unique structural architecture endows PtpB with functional and regulatory properties distinct from both its mycobacterial counterpart PtpA and human PTPs. Unlike PtpA, which exhibits a more conserved classical PTP fold and appears to lack an analogous regulatory lid, PtpB’s dynamic lid and UIM-like region provide a specialized, ubiquitin-dependent activation mechanism crucial for its lipid phosphatase activity (21, 24–26). This specificity highlights PtpB’s unique triple-specificity profile, as it can dephosphorylate both protein and lipid substrates, which are capabilities not found in the mainly tyrosine-specific PtpA (8). Moreover, although the catalytic CX5R motif is conserved, PtpB’s overall fold and active-site pocket topology diverge significantly from those of human PTPs, such as PTP1B (19). Collectively, these structural distinctions delineate unique molecular interfaces that are being harnessed for the design of inhibitors aimed at selectively disrupting PtpB’s virulence functions without cross-reacting with host phosphatases.

In summary, PtpB’s conserved catalytic core, dynamically regulated two-helix lid, and UIM-like ubiquitin-binding region form the basis for its substrate versatility and tightly controlled phosphatase activity, laying the molecular foundation for its role in Mtb virulence and the sabotage of host defense. The functional importance of these structural features is directly supported by genetic evidence: deletion of PtpB in Mtb significantly impairs bacterial survival in macrophages and reduces virulence in animal infection models, confirming that the phosphatase activity enabled by its unique fold is essential for persistence within the host (18). Consequently, PtpB represents an attractive target for the structure-guided development of selective inhibitors as a novel therapeutic strategy against tuberculosis.

## 3 PtpB-mediated dephosphorylation of PI4P and PI(4,5)P2 inhibits GSDMD-dependent pyroptosis

**Figure 2:**
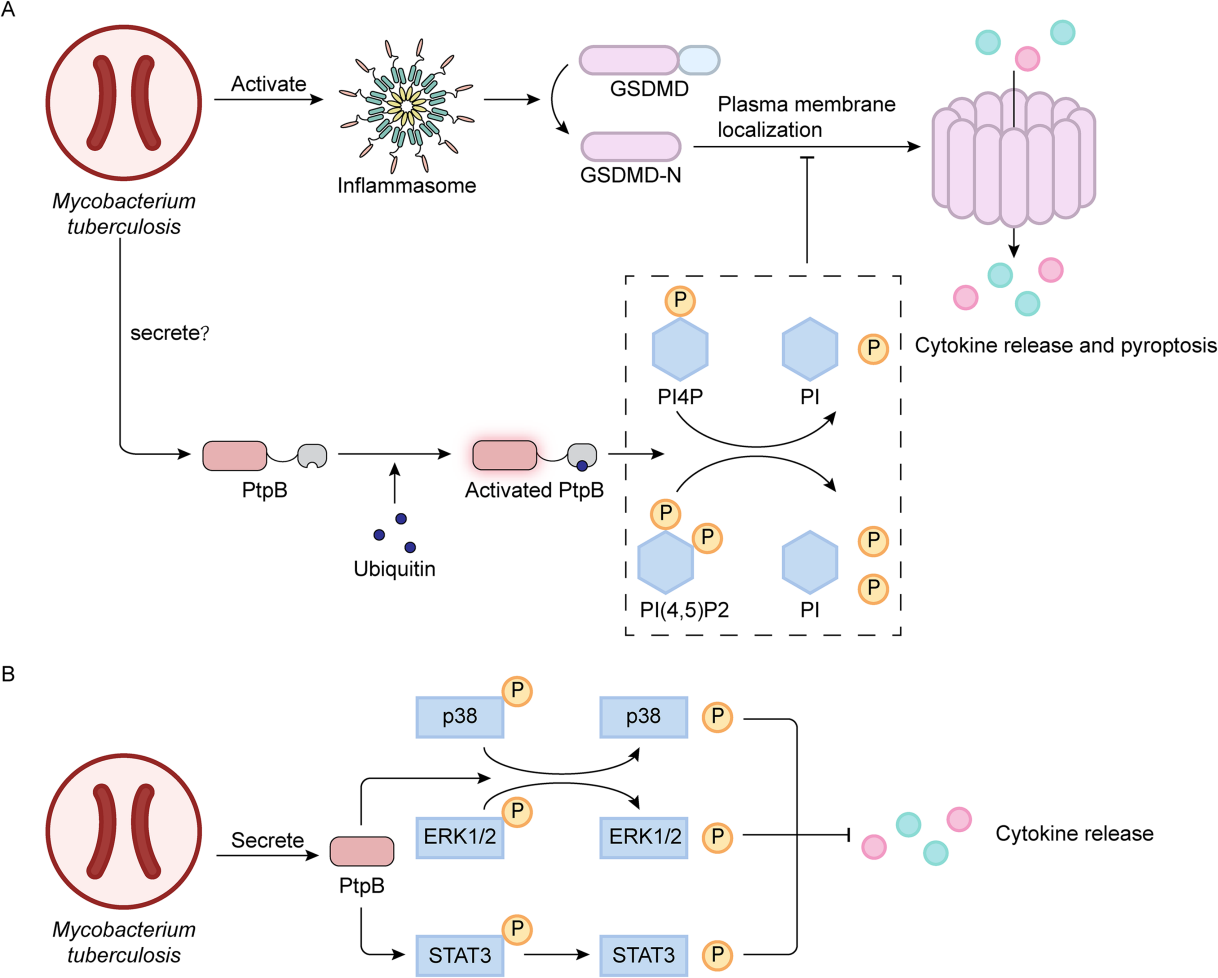
Suggested mechanisms of PtpB-mediated immune suppression. **(A)** PtpB inhibits pyroptosis by hijacking host ubiquitin to alter the composition of plasma membrane phospholipids. Upon secretion into the host cytosol, PtpB binds to mono-ubiquitin via its UIM-like region, triggering an allosteric conformational change that opens the catalytic site. Activated PtpB then dephosphorylates plasma membrane phosphoinositides PI4P and PI(4,5)P2, removing the lipid docking sites necessary for the N-terminal fragment of GSDMD-N to localize to the membrane. This prevents GSDMD-N pore formation, blocks pyroptosis, and enables Mtb to survive within the infected macrophage. **(B)** PtpB facilitates Mtb immune evasion by dephosphorylating host signaling proteins. It directly dephosphorylates p38 and ERK1/2 in the MAPK pathway, as well as STAT proteins in the JAK-STAT pathway, thereby reducing the expression of pro-inflammatory cytokines such as TNF-α, IL-6, and IL-1β, and promoting Mtb immune evasion.

Upon Mtb infection, host innate immunity frequently activates the inflammasome, a multiprotein complex that leads to the cleavage of GSDMD by inflammatory caspases (27). The N-terminal fragment of GSDMD (GSDMD-N) subsequently forms pores in the plasma membrane, culminating in pyroptosis, a lytic form of cell death that eliminates intracellular niches for pathogens and alerts the immune system (28, 29). A crucial step in this process is the localization of GSDMD-N to the plasma membrane, which relies on its specific affinity for phosphoinositide lipids, particularly PI4P and PI(4,5)P2 (27). To counter this host defense, Mtb employs the secreted phosphatase PtpB. As previously described, PtpB must first be activated through its interaction with host mono-ubiquitin (9, 17). Once activated at the host membrane, Chai et al. found that PtpB dephosphorylates PI4P and PI (4,5)P2. By removing these essential lipid signals, PtpB effectively disrupts the membrane localization of GSDMD-N. As a result, pore formation, the release of inflammatory cytokines, and pyroptosis are all inhibited, allowing Mtb to persist within the host cell (**Figure 2A**).

The biological importance of this pathway has been directly demonstrated using genetic and pharmacological approaches. A PtpB mutant lacking phosphatase activity (C160S) fails to block pyroptosis, indicating that the catalytic function of PtpB is essential for this immune evasion mechanism (9). Furthermore, macrophages infected with *PtpB*-deficient Mtb exhibit increased plasma membrane localization of GSDMD-N, enhanced release of IL-1β, and reduced bacterial survival compared to cells infected with wild-type Mtb, as reported by Chai et al. (9). Consistent with these findings, pharmacological inhibition of PtpB using a selective small-molecule inhibitor restores pyroptosis in infected macrophages, leading to enhanced clearance of intracellular Mtb (30). Collectively, these studies validate PtpB as a key suppressor of host pyroptosis and support the therapeutic potential of targeting this phosphatase. The potential impact of PtpB-mediated depletion of PI4P and PI(4,5)P2 on other host membrane-associated processes during Mtb infection remains to be investigated.

The discovery not only uncovers a novel immune evasion mechanism but also paves the way for new avenues in inhibitor design. Targeting the unique ubiquitin-binding interface (UIM-like region) or the lipid phosphatase activity of PtpB could lead to the development of highly specific compounds that disrupt this pathway without impacting host phosphatases. This strategy is particularly promising for combating drug-resistant TB, as it directly counteracts a key virulence mechanism rather than targeting essential bacterial growth pathways.

## 4 PtpB interferes with multiple immune signaling pathways through its phosphatase activity

Beyond its role in inhibiting pyroptosis, PtpB utilizes its phosphatase activity to dephosphorylate key signaling molecules across several critical immune pathways (31). PtpB directly targets the MAPK pathways by dephosphorylating the p38 and ERK1/2 kinases, which have been validated as physiological substrates through *in vitro* and *in vivo* assays (31–33). This action disrupts the downstream signaling cascades that would normally trigger the release of pro-inflammatory cytokines, including TNF-α and IL-6 (31–33). Notably, PtpB not only diminishes phosphorylation levels of these MAPKs but also interferes with the nuclear translocation of activated ERK1/2, thus inhibiting the transcription of immune-related genes within the nucleus (32). The suppression of these signaling molecules at multiple levels substantially undermines the host’s ability to mount an effective inflammatory response against Mtb, aiding in bacterial immune evasion and persistence within the host. Additionally, PtpB targets components of the JAK-STAT signaling pathway, particularly affecting the phosphorylation and subsequent dimerization of STAT3 proteins (32). By disrupting this pathway, PtpB further impairs the expression of pro-inflammatory cytokines (**Figure 2B**). This multi-faceted approach to immune suppression ensures that various aspects of host defense are compromised concurrently. By coordinately disrupting multiple essential immune signaling pathways, PtpB emerges as a critical virulence factor, enabling Mtb to establish and maintain a persistent infection within host immune cells.

## 5 Inhibitors of PtpB in anti-tuberculosis development

The critical role of PtpB in Mtb virulence has established it as a compelling target for novel host-directed therapies, motivating significant research into the development of specific PtpB inhibitors (**Table 1**). These inhibitors are primarily divided into two main categories, each with distinct chemical scaffolds and mechanisms of action.

**Table 1:** Summary of PtpB inhibitors.

Inhibitor	Source/category	Mechanism of action	Evidence type	Limitation	Reference
Brunsvicamides B and C	Natural product (Cyanobacterium *Tychonema* sp.)	Selective inhibitors of PtpB	*In vitro*: IC_50_ for Brunsvicamide B = 7.3 µM, for Brunsvicamide C = 8.0 µM.	Cellular and *in vivo* activity remains unexplored.	(34)
Indolo[2,3-a]quinolizidine derivative	Synthetic (Biology-Oriented Synthesis, BIOS)	–	*In vitro*.	Cellular and *in vivo* activity remains unexplored; the mechanism unknown.	(35)
Compound 6-hydroxy-2-phenyl-3((3-trifluoromethyl)phenyl)benzofuran-5-carboxylic acid (4g)	Natural product derivative (Benzofuran salicylic acid scaffold)	Selective inhibitor of PtpB	*In vitro*: IC_50_ = 38 nM. In cellular: Restores ERK1/2 and p38 activity and IL-6 production.	*In vivo* activity remains unexplored.	(36)
Fusarielin M	Natural product (Marine-derived fungus Fusarium graminearum SYSU-MS5127)	Competitive inhibitor of PtpB	*In vitro*: IC_50_ = 1.05 ± 0.08 µM, K_i_ = 1.03 ± 0.39 µM. In cellular: Restores ERK1/2 and p38 activity and IL-6 production.	*In vivo* activity remains unexplored.	(37)
Longidiacid A and Longichalasin B	Natural product (Deep-sea-derived fungus Diaporthe longicolla FS429)	–	*In vitro*: At 50 µM, inhibit 35.4% (Longidiacid A) and 53.5% (Longichalasin B) of PtpB enzyme activity.	Low inhibitory activity; cellular and *in vivo* activity remains unexplored; the mechanism unknown	(38)
Kuwanol E	Natural product (Morus nigra cell cultures)	Competitive inhibitor of PtpB	*In vitro*: IC_50_ = 1.9 ± 0.5 µM, K_i_ = 1.6 ± 0.1 µM.	Cellular and *in vivo* activity remains unexplored.	(20)
ESA and DTP	Natural product (Virtual screening of the ZINC database)	Predicted to bind the active site of PtpB via hydrogen bonds and hydrophobic interactions	*In silico*: 3D-QSAR prediction: pIC_50_ = 1.459 (ESA) and 1.677 (DTP).	Only computational predictions; lack of experimental validation (enzymatic, cellular, or *in vivo*).	(39)
(+)-aS-Alterporriol C	Natural product (Mangrove Endophytic Fungus, Alternaria sp. SK11)	–	*In vitro*: IC_50_ = 8.70 nM.	Cellular and *in vivo* activity remains unexplored; the mechanism unknown.	(40)
(±)-Asperlone A, (±)-Asperlone B and (−)-Mitorubrin	Natural product (Mangrove Endophytic Fungus, Aspergillus sp. 16-5C)	–	*In vitro*: IC_50_ = 4.24 ± 0.41 µM ((±)-Asperlone A), 4.32 ± 0.60 µM ((±)-Asperlone B) and 3.99 ± 0.34 µM ((−)-Mitorubrin).	Cellular and *in vivo* activity remains unexplored; the mechanism unknown.	(41)
Diaporisoindole A and Tenellone C	Natural product (Mangrove Endophytic Fungus, Diaporthe sp. SYSU-HQ3)	–	*In vitro*: IC_50_ = 4.2 µM (Diaporisoindole A) and 5.2 µM (Tenellone C).	Cellular and *in vivo* activity remains unexplored; the mechanism unknown.	(42)
OMTS ((oxalylamino-methylene)-thiophene sulfonamide)	Synthetic (Based on the oxamic acid scaffold)	Competitive inhibitor of PtpB	*In vitro*: IC_50_ = 440 ± 50 nM.	Cellular and *in vivo* activity remains unexplored.	(19)
Sulfonamide (S1-S6) and Acetamide (N1-N5) derivatives	Synthetic (Pyrimidine derivatives)	Predicted to bind the active site of PtpB via hydrogen bonds and π-π stacking	*In silico*.	Only computational predictions; lack of experimental validation (enzymatic, cellular, or *in vivo*).	(43)
γ-Lactone derivative	Natural product (Virtual screening)	Predicted to bind the active site of PtpB via hydrogen bonds	*In vitro*: IC_50_ = 31.1 µM.	Cellular and *in vivo* activity remains unexplored.	(44)
4-(3′,5′-Dichloro-4′-hydroxy-3-biphenyl)-5-methylisoxazole-3-carboxylic Acid	Synthetic (Isoxazole-based compounds)	Selective inhibitor of PtpB	*In vitro*. Cellular. *In vivo*.	Low inhibitory activity *in vitro*.	(30)
Isoxazole-salicylate derivative	Synthetic (Isoxazole-based compounds)	Competitive inhibitor of PtpB	*In vitro*: IC_50_ = 7 µM, K_i_ = 1.5 µM. Cellular.	*In vivo* activity remains unexplored.	(45)
F1S-6C-W11	Synthetic (Click chemistry strategy)	Competitive inhibitor of PtpB	*In vitro*: IC_50_ = 0.64 ± 0.09 µM, K_i_ = 0.15 ± 0.01 µM.	Cellular and *in vivo* activity remains unexplored.	(46)
L5B47	Synthetic (Click chemistry strategy)	Non-competitive inhibitor of PtpB	*In vitro*: IC_50_ = 160 ± 10 nM, K_i_ = 162 ± 10 nM.	Cellular and *in vivo* activity remains unexplored.	(47)
Indolin-2-on-3-spirothiazolidinone	Synthetic (Screening of a compound library)	Competitive inhibitor of PtpB	*In vitro*: IC_50_ = 0.32 ± 0.2 µM, K_i_ = 250 ± 10 nM.	Cellular and *in vivo* activity remains unexplored.	(48)
Pyrrole-salicylic acid derivative	Synthetic (organocatalytic MCR library)	Selective inhibitor of PtpB	*In vitro*: IC_50_ = 1.5 µM.	Cellular and *in vivo* activity remains unexplored.	(49)
I-A09	Synthetic (Click chemistry strategy)	Non-competitive inhibitor of PtpB	*In vitro*: IC_50_ = 1.26 ± 0.22 µM; K_i_ = 1.08 ± 0.06 µM. Cellular: Restores ERK1/2 and p38 activity and IL-6 production.	*In vivo* activity remains unexplored.	(33)
1,2,3-triazole-tethered 4H-pyrano[2,3-d]pyrimidine-D-glucose conjugate	Synthetic (Click chemistry strategy)	Competitive inhibitor of PtpB	*In vitro*: IC_50_ = 1.56 ± 0.21 µM, K_i_ = 1.10 ± 0.09 µM.	Cellular and *in vivo* activity remains unexplored.	(50)
hiobarbiturate derivative	Synthetic (Virtual screening)	Non-competitive inhibitor of PtpB	*In vitro*: IC_50_ = 22.4 ± 2.5 µM, K_i_ = 24.7 ± 0.8 µM. Cellular.	*In vivo* activity remains unexplored.	(51)
Biphenyl thiobarbiturate derivative	Synthetic (structure-based strategy)	Non-competitive inhibitor of PtpB	*In vitro*: IC_50_ = 1.18 ± 0.11 µM, K_i_ = 1.01 µM. Cellular.	*In vivo* activity remains unexplored.	(52)
N-aryl oxamic acid derivative	Sybthetic (N-aryl oxamic acid derivative)	Competitive inhibitor of PtpB	*In vitro*: IC_50_ = 6.4 ± 0.5 nM, K_i_ = 2.7 ± 0.2 nM. Cellular: Restores ERK1/2 and p38 activity and IL-6 production.	*In vivo* activity remains unexplored.	(53)
Rhodanine derivative	Synthetic (structure-based strategy)	Selective inhibitors of PtpB	*In vitro*: IC_50_ = 0.64 ± 0.04 µM. Cellular.	*In vivo* activity remains unexplored.	(54)

The first major category is natural products and their derivatives (**Table 1**). The cyclic hexapeptides brunsvicamide B and C, produced by the cyanobacterium *Tychonema* sp., represent the first reported peptide inhibitors of PtpB based on enzymatic inhibition assays, providing a novel structural template for the development of anti-tuberculosis drugs (34). However, their cellular activity and *in vivo* efficacy remain to be investigated. A compound library containing indolo[2,3-a]quinolizidine scaffolds was constructed using a Biology-Oriented Synthesis (BIOS) strategy, leading to the identification of a novel class of natural product-derived compound inhibitors that were active in enzymatic assays and served as valuable tool molecules in tuberculosis-related biological research. Their potential as antitubercular leads awaits further validation in cell and animal models (35). Notably, compound 6-hydroxy-2-phenyl-3((3-trifluoromethyl)phenyl)benzofuran-5-carboxylic acid (4g), a derivative based on a natural product core scaffold, and fusarielin M, isolated from the marine-derived fungus *Fusarium graminearum SYSU-MS5127* both demonstrated the ability to restore ERK1/2 and p38 activity and IL-6 production, thus preventing the intracellular growth of Mtb (36, 37). Other compounds, longidiacid A and ongichalasin B, obtained from deep-sea-derived fungus *Diaporthe longicolla FS429*, displayed only weak inhibitory activity against PtpB (38). Additionally, a seminal study identified six natural compounds that inhibit PtpB at low micromolar concentrations (<30 µM), with the most potent being Kuwanol E (K_i_ = 1.6 ± 0.1 µM), a polyhydroxylated Diels-Alder type adduct isolated from Morus nigra cell cultures. The discovery of Kuwanol E and related adducts not only confirmed the therapeutic potential of inhibiting PtpB but also provided valuable chemical scaffolds for developing novel host-directed anti-tuberculosis agents (20). A study developed a 3D QSAR model to screen natural compounds, identifying ESA and DTP as high-affinity binders to the PtpB active site *in silico*, thereby showcasing their potential as anti-tuberculosis therapeutics. However, experimental validation, including enzymatic assays and cellular studies, is required to confirm their anti-tuberculosis efficacy (39). Compounds isolated from mangrove-associated microorganisms, including (+)-aS-Alterporriol C, (±)-asperlone A, (±)-asperlone B, (−)-mitorubrin, diaporisoindole A, and tenellone C have also been shown to inhibit PtpB activity (40–42).

The other major category is artificially synthesized inhibitors (**Table 1**). A landmark achievement in this field was the identification and structural characterization of the competitive inhibitor OMTS. OMTS demonstrates potent and selective inhibition of PtpB, with an IC_50_ of 440 ± 50 nM and more than 60-fold selectivity against a panel of human PTPs (PTP1B, Pac1, Glepp1, and PTPH1) (19). The crystal structure of the PtpB-OMTS complex revealed that inhibitor binding induces significant conformational changes. Specifically, it promotes a large hinge motion in one helix of the lid, forming a hydrophobic hairpin and a channel that leads to the catalytic cysteine. Furthermore, a 30-residue disordered loop folds to form a new helix at the active site. This structural insight highlighted a unique secondary substrate-binding pocket and provided a novel strategy for designing inhibitors that exploit PtpB-specific structural features, thereby achieving selectivity over host phosphatases (19). Building on these findings, a series of innovative compounds, including sulfonamide (S1-S6) and acetamide (N1-N5) derivatives were synthesized, exhibiting significant binding energies (ranging from −46 kcal/mol to −61 kcal/mol) with PtpB. These compounds bind to the same active site as OMTS while forming stable interactions through hydrogen bonding and π-π stacking (43). Referencing the X-ray structure of the PtpB-OMTS complex, γ-lactone derivatives have also been shown to exhibit enzymatic activity against PtpB, establishing a reliable paradigm for discovering novel target inhibitors (44). Additionally, isoxazole-based compounds represent an important class of PtpB inhibitors. The isoxazole head group binds to the active site (P1 pocket) of PtpB, while the salicylate or dichlorophenol groups interact with a unique secondary pocket (P2 pocket), thereby enhancing the inhibitor’s selectivity toward PtpB (30, 45). Using the click chemistry strategy, bidentate compounds such as H16C-W11 and F1S-6C-W11, as well as tridentate compounds like L5B47, can be synthesized. These compounds also exhibit inhibitory activity against PtpB (46, 47). Furthermore, Indolin-2-on-3-spirothiazolidinones were identified as a class of substrate-competitive inhibitors of PtpB, exhibiting remarkable specificity and showing no significant inhibition against six homologous phosphatases (including PtpA and various mammalian phosphatases like PTP1B, SHP-2, PTPN2, h-PTPb, and VHR), even at a concentration of 50 µM (48).

A number of synthetic inhibitors can also enhance their inhibitory activity and target selectivity through the dual-site binding strategy (**Table 1**). A potent and highly selective PtpB inhibitor with an IC_50_ value of 1.5 µM and >50-fold specificity was synthesized through an efficient organocatalytic multicomponent reaction involving pyrrole, formaldehyde, and aniline. This inhibitor operates via a dual-binding mode, targeting both the active site and an adjacent secondary site. The salicylate moiety occupies the PtpB active site, while the aniline-derived fragment binds to a peripheral site, enhancing the affinity for PtpB (49). Another inhibitor, I-A09, shares a similar mechanism of action (33). A recent study synthesized 24 novel conjugated derivatives containing 1,2,3-1H-triazoles with 4H-pyrano[2,3-d]pyrimidine (8a-y); among these, six compounds (8g, 8t, 8u, 8v, 8x, 8y) exhibited significant inhibitory activity against PtpB, with IC_50_ values ranging from 1.56 to 9.52 µM. The most potent compounds, 8v, 8x, and 8y, were identified as competitive inhibitors, providing candidate compounds for developing highly effective PtpB inhibitors (50). Employing a structure-based virtual screening strategy, compounds containing a thiobarbiturate scaffold were identified and subsequently optimized through structural modifications, such as the introduction of a biphenyl fragment and fine-tuning of substituent positions, leading to the development of more potent PtpB inhibitors (51, 52). Recent medicinal chemistry efforts have further expanded the repertoire with novel synthetic classes, such as N-aryl oxamic acid derivatives, which demonstrate improved potency and selectivity profiles. Among this series, compound 4t exhibited a K_i_ of 2.7 nM for PtpB and over 4500-fold selectivity for PtpB compared to a panel of 25 mammalian PTPs (53). Rhodanine derivatives have also shown inhibitory activity against PtpB in enzymatic assays, with some compounds demonstrating dual inhibition of both PtpA and PtpB, thereby contributing to their anti-tuberculosis effects. However, their *in vivo* efficacy remains to be characterized (54).

Despite these promising advancements, the development of clinically viable PtpB inhibitors faces substantial challenges. A significant hurdle is the inherent difficulty of converting potent *in vitro* inhibitors into compounds with suitable cell permeability and oral bioavailability, as the positively charged, hydrophilic catalytic pocket tends to attract polar, negatively charged molecules that poorly traverse cell membranes (55). Furthermore, demonstrating sufficient target selectivity in the complex cellular environment to avoid off-target effects remains a critical challenge. Ultimately, to harness their full therapeutic potential, the most viable strategy for PtpB inhibitors will likely involve combination therapy with existing antibiotics. However, identifying optimal combination regimens to shorten treatment duration or overcome bacterial persistence will necessitate extensive preclinical and clinical investigations to validate synergistic efficacy and safety.

## 6 Conclusions and prospects

The multifaceted immune suppression exerted by PtpB, which targets both key signaling proteins and plasma membrane lipids, reinforces its status as a critical virulence factor. Its unique structural attributes, particularly the ubiquitin-activated dynamic lid, not only enable this functional versatility but also differentiate it from host phosphatases, providing a compelling rationale for its development as a drug target. However, translating this mechanistic promise into clinical reality necessitates a thorough assessment of both the advances and the unresolved challenges in the field, especially in light of the rising prevalence of drug-resistant strains.

To effectively translate this potential target into clinical applications, several key challenges must be addressed, and foundational knowledge must be expanded. Future investigations should first aim to clarify the precise spatial and temporal regulation of PtpB activity within host cells, elucidating how its secretion, ubiquitin-dependent activation, and substrate selection are coordinated during infection. Second, despite the strong proof-of-concept provided by potent and selective inhibitors such as Kuwanol E and OMTS, as highlighted in recent analyses, the journey from validating PtpB as a target to developing clinically viable inhibitors entails navigating common hurdles associated with phosphatase-targeted drugs, including poor cell permeability and low oral bioavailability. Innovative medicinal chemistry approaches will be essential, potentially focusing on allosteric sites such as the regulatory lid or the ubiquitin-binding interface. Third, the potential for Mtb to develop resistance to PtpB inhibitors is an important yet underexplored consideration, necessitating studies into possible bacterial escape mechanisms. Finally, the therapeutic utility of PtpB inhibition must be rigorously validated in more complex physiological models. The most feasible clinical application is likely to involve combination therapy with existing antibiotics; thus, identifying synergistic partners capable of shortening treatment duration or eradicating persistent bacterial populations will be critical. Progress in these areas requires a coordinated interdisciplinary effort. The continued exploration of PtpB, with the aim of translating mechanistic insights into a novel class of host-directed therapeutics, remains a high priority in tuberculosis research.
